# Changes in the Functional Brain Network of Children Undergoing Repeated Epilepsy Surgery: An EEG Source Connectivity Study

**DOI:** 10.3390/diagnostics11071234

**Published:** 2021-07-09

**Authors:** Giulia Iandolo, Nitish Chourasia, Georgios Ntolkeras, Joseph R. Madsen, Christos Papadelis, Ellen Grant, Phillip L. Pearl, Fabrizio Taffoni, Eleonora Tamilia

**Affiliations:** 1 Fetal Neonatal Neuroimaging and Developmental Science Center, Department of Pediatrics, Boston Children’s Hospital, Boston, MA 02115, USA; giulia96.iandolo@gmail.com (G.I.); Georgios.Ntolkeras@childrens.harvard.edu (G.N.); Ellen.Grant@childrens.harvard.edu (E.G.); 2 Advanced Robotics and Human-Centred Technologies-CREO Lab, School of Engineering, University Campus Bio-Medico di Roma, 00128 Rome, Italy; f.taffoni@unicampus.it; 3 Division of Epilepsy and Clinical Neurophysiology, Boston Children’s Hospital, Harvard Medical School, Boston, MA 02115, USA; nitish.chourasia@childrens.harvard.edu (N.C.); Phillip.Pearl@childrens.harvard.edu (P.L.P.); 4 Health Science Center, Department of Pediatrics and Neurology, Division of Peds Neurology, Le Bonheur Children’s Hospital, College of Medicine, University of Tennessee, Memphis, TN 38103, USA; 5 Division of Newborn Medicine, Boston Children’s Hospital, Harvard Medical School, Boston, MA 02115, USA; Christos.Papadelis@cookchildrens.org; 6 Epilepsy Surgery Program, Department of Neurosurgery, Boston Children’s Hospital, Harvard Medical School, Boston, MA 02115, USA; Joseph.Madsen@childrens.harvard.edu; 7 Jane and John Justin Neurosciences Center, Cook Children’s Health Care System, Fort Worth, TX 76104, USA; 8 Department of Bioengineering, University of Texas at Arlington, Arlington, TX 76019, USA

**Keywords:** electroencephalography, epilepsy, epilepsy surgery, functional connectivity, brain networks, children, source imaging, neuroimaging, clinical neurophysiology

## Abstract

About 30% of children with drug-resistant epilepsy (DRE) continue to have seizures after epilepsy surgery. Since epilepsy is increasingly conceptualized as a network disorder, understanding how brain regions interact may be critical for planning re-operation in these patients. We aimed to estimate functional brain connectivity using scalp EEG and its evolution over time in patients who had repeated surgery (RS-group, *n* = 9) and patients who had one successful surgery (seizure-free, SF-group, *n* = 12). We analyzed EEGs without epileptiform activity at varying time points (before and after each surgery). We estimated functional connectivity between cortical regions and their relative centrality within the network. We compared the pre- and post-surgical centrality of all the non-resected (untouched) regions (far or adjacent to resection) for each group (using the Wilcoxon signed rank test). In alpha, theta, and beta frequency bands, the post-surgical centrality of the untouched cortical regions increased in the SF group (*p* < 0.001) whereas they decreased (*p* < 0.05) or did not change (*p* > 0.05) in the RS group after failed surgeries; when re-operation was successful, the post-surgical centrality of far regions increased (*p* < 0.05). Our data suggest that removal of the epileptogenic focus in children with DRE leads to a gain in the network centrality of the untouched areas. In contrast, unaltered or decreased connectivity is seen when seizures persist after surgery.

## 1. Introduction

Epilepsy is widely regarded as a brain network disorder, corroborated by an increasing body of evidence demonstrating alterations in an inter-regional anatomofunctional relationship among brain areas in the epileptic brain [1,2,3,4,5]. Consequently, clinical seizures are hypothesized to be secondary to involvement of large-scale synchronized brain networks [6,7]. This understanding of seizures and epileptic networks has been often elaborated in a network-level perspective by using functional connectivity (FC), a concept that is based on the assumption that the extent to which different brain areas are functionally connected depends on their level of synchronous temporal activity [8]. FC analysis in epilepsy has been investigated via resting-state functional magnetic resonance imaging (fMRI) [9,10,11,12,13,14], intracranial electroencephalogram (iEEG) [15,16], scalp electroencephalogram (EEG) [17,18,19], or magnetoencephalography (MEG) [20,21,22].

In patients with focal drug-resistant epilepsy (DRE), the understanding of epileptogenic network connectivity has therapeutic relevance, especially regarding epilepsy surgical evaluation. Underpinning this process is the formulation of a network hypothesis regarding identification of structures within the epileptogenic networks important in the generation of seizures [23]; however, despite elaborate pre-surgical evaluation (consisting of scalp and intracranial EEG, MRI, functional MRI (fMRI), PET, magnetoencephalography (MEG), and neuropsychological testing), nearly one-third of patients suffer failure of first resective epilepsy surgery with continued seizures [24,25]. Some of these patients may still benefit from repeat surgery, although defining such a re-operation plan after failed surgical resection can be challenging [26,27,28,29]. Thus, exploration of additional noninvasive techniques that can complement the pre-surgical evaluation test battery would be valued clinical tools in such complex cohorts.

There are few studies that have reviewed patients undergoing repeat epilepsy surgery in the pediatric age group [26,27,28], evaluating outcomes and their predictors after re-operation using clinically available pre-surgical data (such as etiology, pathology, type and site of resection, age, time between surgeries, and so on). There is a paucity of research studies investigating new analytic approaches in this population, so as to improve or complement the clinically established pre-surgical evaluation tests. To this effect, deconstructing the functional brain network and evaluating its connectivity changes after surgery is a new approach in such a cohort. It can be speculated that the quantification of brain network characteristics may aid in providing supplemental information for surgical planning in these cases.

Among all the available non-invasive modalities that can be used to quantify FC, scalp EEG stands out as an ideal tool owing to its frequent and universal usage in clinical management of patients with epilepsy. Besides its large availability, scalp EEG provides high temporal resolution (millisecond time scale typically) that allows the investigation of brain networks at different frequency bands as opposed to fMRI. Although scalp EEG suffers from lower spatial resolution than its invasive counterpart (stereotactic EEG or electrocorticography) or fMRI, it allows the estimation of whole-brain functional networks, especially if combined with the spatial information of the individual MRI (performing what is called electric source imaging or ESI) [30,31,32,33,34]. Despite the contribution of aberrant brain connectivity in generation of seizures being increasingly recognized, it remains to be explored whether connectivity changes following a surgical brain resection can be quantified via routine scalp EEG and linked to seizure occurrence. In addition, even though pre-surgical aspects of the FC brain network have been extensively studied [2,12,35,36,37,38,39,40], there is little knowledge about how FC evolves before and after epilepsy surgery.

In this study, we study serial scalp EEGs from children with DRE who underwent repeat focal epilepsy surgery with the aim of (i) quantifying surgery-related changes in the children’s functional brain network (i.e., evolution of their brain network organization) and (ii) identifying whether such brain network evolution differs depending on the surgical outcome. For this latter point, we also study FC evolution in children with DRE who underwent a single successful surgery. We perform FC analysis at the source level (between cortical regions) using scalp EEG measurements collected during sleep before and after each surgery along with the individual patient’s MRI scans. We compute the FC of the brain during the interictal state using EEG epochs free of any interictal epileptiform discharges.

## 2. Materials and Methods

### 2.1. Subjects

We studied children with DRE who underwent focal epilepsy surgery at the Epilepsy Center of Boston Children’s Hospital (BCH) between November 2013 and March 2019 and had a minimum duration of follow-up of 1 year since last surgery. We studied two groups of patients: (1) repeated surgery group, i.e., patients who had two focal resective surgeries (because of seizure recurrence after the first one); (2) seizure-free group, i.e., patients who had one focal resective surgery followed by good outcome (namely, who were Engel 1 after 12 months from surgery). Inclusion criteria for both groups were: (i) availability of sleep EEG data prior and after each surgery; (ii) availability of both pre-surgical and post-surgical MRIs. Exclusion criteria were: (i) hemispherectomy or corpus callosotomy (non-focal resections); (ii) absence of at least 1 min of non-REM sleep in the EEG recordings that was free of artifacts and epileptic spikes; (iii) unsatisfactory quality of the MRIs for coregistration and delineation of resection. The study protocol received approval by the BCH Institutional Review Board (IRB-P00035192, PI: Tamilia), which waived the need for written informed consent due to the study’s retrospective character. Post-surgical outcome following each surgery was evaluated by a pediatric epileptologist from the most recent follow-up visit using the Engel scale, as is clinical practice at our institution, dichotomized into good outcome (Engel 1) and poor outcome (Engel ≥ 2).

### 2.2. Scalp EEG Data

**Figure 1:**
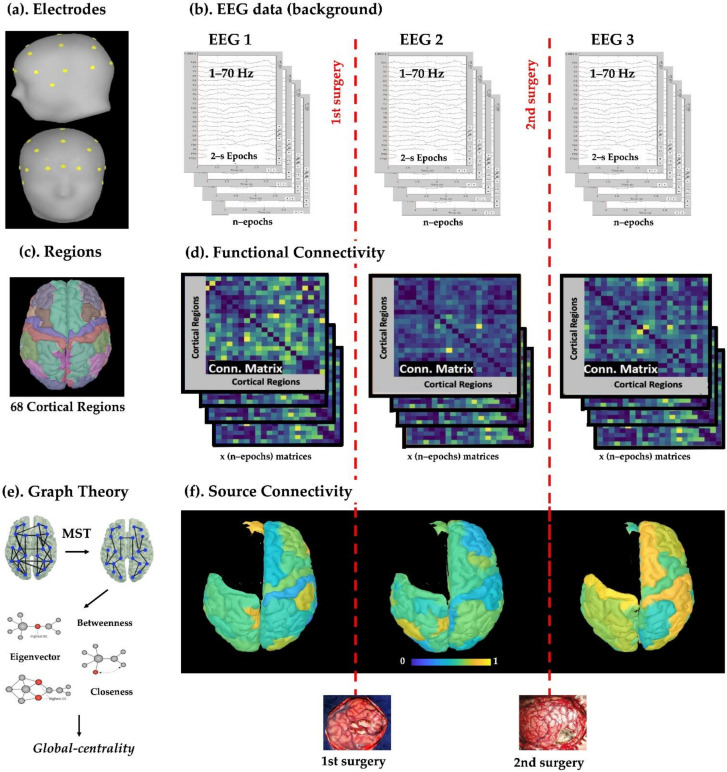
Outline of the data analysis process. (**a**) We retrieved conventional EEG data and the patient’s MRI at the time of the EEG (to build the head model), on which electrodes were positioned according to the international 10/20 system); (**b**) interictal sleep EEG data without interictal discharges and artifacts were selected (“background activity”). We analyzed three EEGs per patient for the repeated surgery group (EEG1—before first surgery; EEG2—between surgeries; EEG—after second surgery) and two EEGs per patient for the seizure-free group (EEG1—before surgery; EEG2—after surgery). These EEG data were segmented into multiple 2 s epochs. (**c**) Each patient’s cortical surface was parcellated into 68 regions of interest (ROIs) defined by the Desikan–Killiany atlas. (**d**) From each EEG epoch, a functional connectivity matrix was computed (amplitude envelope correlation method). (**e**) A graph and its minimum spanning tree (MST) were then built from each matrix, then three centrality measures (betweenness, closeness, and eigenvector) were extracted, normalized, and averaged, obtaining a unique comprehensive measure of relative centrality (“global centrality”). (**f**) The global centrality was estimated for each cortical ROI (average of all epochs) at each time point (before first surgery, between surgeries, after second surgery). We report here an example of global centrality across all cortical ROIs for patient #2, where yellow and blue colors indicate high and low values, respectively.

All patients underwent conventional video-EEG recording at Boston Children’s Hospital (BCH) using the standard clinical EEG setup with 19 electrodes, plus two additional frontotemporal leads (FT9 and FT10) and four optional electrodes in some cases (F9, F10, P9, P10). Data were recorded using the XLTEK EMU40 system (Natus Medical Inc., Pleasanton, CA, USA) with a sampling rate in the 200–1024 Hz range. Individual leads (gold cups) were placed according to the international 10/20 system (Figure 1a). Impedances were kept below 10 KΩ during the entire recording session. Reference and ground electrodes were placed in frontocentral areas (near FC1 and FC2, respectively). EEG data were filtered using a notch filter at 60 Hz (second order IIR notch filter with zero-phase lag) and a 1–70 Hz band-pass filter (Figure 1b). Band-pass filtering was performed using Brainstorm software; this implements an even-order linear phase FIR filter based on a Kaiser window design, which is then made to be zero-phase and zero-delay by shifting the sequence backward in time.

We retrieved three EEGs per patient for the repeated surgery group, as shown in Figure 1b: (1) before first surgery; (2) between surgeries; (3) after second surgery. Two EEGs per patient were analyzed for the seizure-free group, before and after surgery. As part of the EEG monitoring at BCH, pediatric neurophysiologists extracted multiple 5–10 min segments of interictal data from the EEG recordings (long-term monitoring or routine EEG). For the purpose of this study, a pediatric clinical neurophysiologist (N.C.), blinded to the resection and the surgical outcome, retrospectively reviewed these segments and selected for each patient 3–5 min of data from stage N1 or N2 sleep, without including major artifacts, technical disruptions, ictal events, or continuous interictal discharges. A second independent neurophysiologist (P.L.P.) reviewed the same segments, blinded to the first reading, to confirm sleep stage and absence of ictal events. Spikes were then identified through visual inspection by two independent readers (N.C. and G.N.) on a bipolar montage. In case of disagreement, a third senior epileptologist was available (P.L.P). Epochs of 2000 ms around each spike were excluded from further analysis (in order to analyze background activity). Any activity related to an excessive electrode artifact was also marked and excluded. The signal space projection (SSP) technique was used to reject external disturbances due to cardiac events detected by electrocardiogram (ECG) recordings when present. Finally, clean background EEG data (free of artefacts and epileptiform activity) were divided into 2 s epochs (see Figure 1b).

### 2.3. Cortical Parcellation and Regions of Interest

We retrieved the pre-operative and post-operative MRIs for each patient. As part of their routine pre- and post-surgical evaluation, a T1-weighted volumetric MRI was acquired, typically a magnetization-prepared rapid gradient echo (MPRAGE). Pre-operative and post-operative MRIs were pre-processed in FreeSurfer [41] (or Infant Freesurfer [42], when the first failed) to segment brain structures and extract the pial surface (cortex). As part of this process, each patient’s cortical surface was mapped to the Desikan–Killiany brain atlas [43], which assigns each neocortical vertex to one of the 68 areas based on gyral morphology. Through this atlas, we automatically defined the 68 regions of interest (ROIs) for the following whole-brain connectivity analysis.

### 2.4. Estimation of Source Activity and Cortical Parcellation

**Figure 2:**
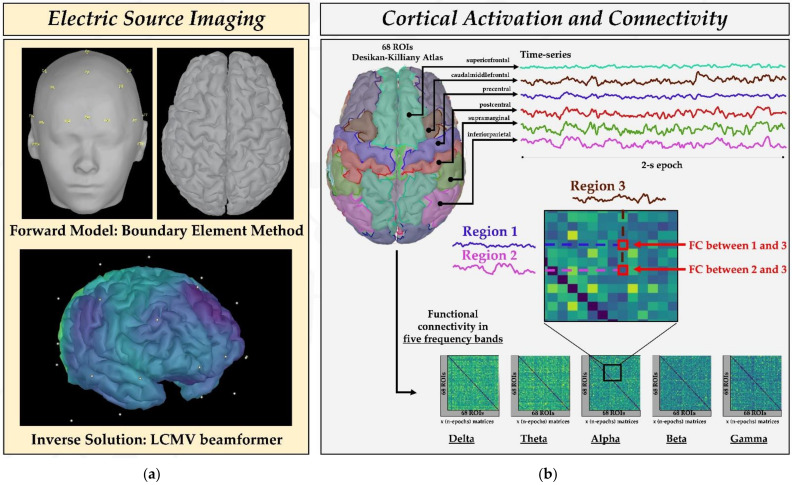
Source connectivity analysis. (**a**) To estimate the cortical activity from EEG data, we performed electric source imaging (ESI). A 3-layer boundary element method (BEM) was applied to solve the forward problem (i.e., compute the head model) after extracting the 3D head mask and cortical surface (**top left**) from each patient’s MRI. Electrodes were positioned on the head based on the 10–20 system. A linearly constrained minimum variance (LCMV) beamformer was used to solve the inverse problem, which generated a cortical activation map for each 2 s epoch (**bottom left**). (**b**) We estimated the activity of each cortical region of interest (ROI), obtaining a time series for each ROI. Functional connectivity between these regions was estimated for five frequency bands (δ (2–4 Hz); θ (5–7 Hz); α (8–12 Hz); β (13–29 Hz); γ (30–70 Hz)). Each element of the connectivity matrix indicates how much an ROI is connected to another in that 2 s epoch.

ESI was performed on EEG data using Brainstorm [44], as illustrated in Figure 2a. For this purpose, we constructed a realistic head model using OpenMEEG software after manually co-registering the EEG channel locations with the patient’s MRI based on the 10–20 system (top of Figure 2b) [31,45,46].

For the head model, we used a three-layer (scalp, outer skull, and inner skull) boundary element method (BEM). Source space was constrained to the cortex, which was composed of ~15,000 vertices per patient. We used a linearly constrained minimum variance (LCMV) beamformer to estimate brain activity within the source space [47]. Data covariance was computed from the EEG epochs, whereas identity matrix was used for noise covariance.

We obtained a source activation map (covering all cortical vertices) for each 2 s epoch (bottom of Figure 2a). The beamformer output was used to estimate the neural activity of each ROI, defined via the Desikan–Killiany atlas, by computing their mean activation (mean across cortical vertices). For each of these regions we obtained a time series (cortical activation over time), as shown in Figure 2b.

### 2.5. Classification of ROIs Based on the Resection

**Figure 3:**
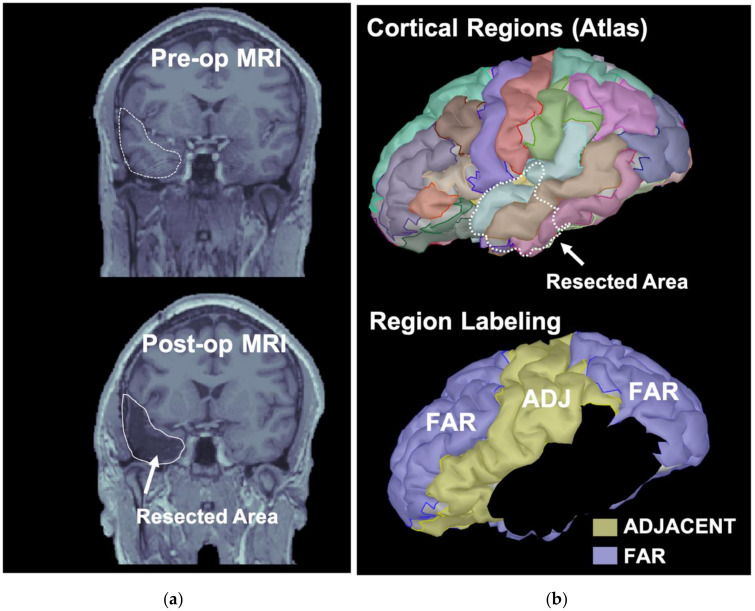
Resection and cortical region labeling. (**a**) Pre-operative (**top**) and post- operative (**bottom**) MRIs are displayed with the resected area (white outline). (**b**) Resected area (left temporal lobe in this patient) is displayed on top of the 68 cortical ROIs. The ROIs overlapping (even partially) with the resected areas were identified (black regions, bottom). All other ROIs were labelled as either adjacent to the resection (ADJ, in yellow) if within 10 mm from the closest margin of the resected area or far from the resection otherwise (in purple).

Resection was drawn manually in Brainstorm slice-by-slice after co-registration of the pre- and post-operative MRIs (see Figure 3a). We computed the distance of each cortical ROI from the resection as the minimum Euclidean distance between all their cortical vertices. Regions that had <10% of their vertices resected were regarded as untouched during surgery (remaining cortical regions). All other ROIs were considered as overlapping with the resection and were excluded from further analysis. We excluded partially resected ROIs from our study because: (i) their automated parcellation on the post-operative cortex could be affected by errors due to the abnormal brain structure; (ii) the observed post-surgical connectivity changes could be significantly biased by the actual structural changes of these regions (due to partial resection). Given the limited sample size of our cohort, we could not control for these factors.

The untouched regions in the same hemisphere of the resection that had a distance from the resection lower or equal to 10 mm were labeled as “adjacent”; all other remaining regions were labeled as “far” (see example in Figure 3b).

### 2.6. Functional Connectivity Analysis

**Figure 4:**
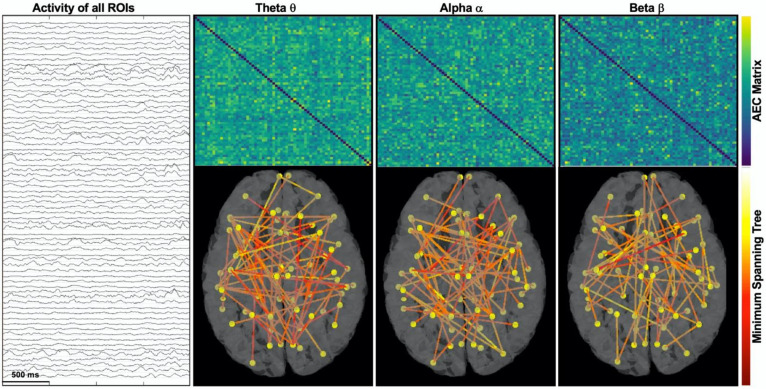
Example of source connectivity and minimum spanning tree in three frequency bands. Left panel shows one 2 s epoch of the background source activity (68 signals), whereby each signal corresponds to the reconstructed activity (via ESI) of each cortical ROI across the whole brain. From these reconstructed cortical signals, we estimated 5 connectivity matrices (via AEC method) and their minimum spanning tree (MST) for each frequency band. Here, we show matrices and graphs (MST) for theta, alpha, and beta bands (from the 2-s epoch displayed on the left), whereby each node (yellow dots) corresponds to an ROI and each edge (link or connection) is color-coded based on its cost (inverse of AEC value). Network centrality measures were estimated from each MST and then averaged across all analyzed epochs.

To estimate FC, we computed the orthogonalized amplitude envelope correlation (AEC) [48,49] between each pair of ROIs, which consisted of the linear correlation between orthogonalized, band-limited power envelopes. Orthogonalization was used to compensate the spatial leakage and to remove all shared signals at zero lag between the network nodes. We chose this method based on the study of Colclough et al. [50], which showed that AEC is the most consistent connectivity measure for resting-state studies in source space. AEC was estimated in five frequency bands (Figure 2b): (i) delta δ (1–4 Hz); (ii) theta θ (5–7 Hz); (iii) alpha α (8–12 Hz); (iv) beta β (13–29 Hz); (v) gamma γ (30–70 Hz, low gamma). We computed the AEC for each 2 s epoch and obtained a FC matrix per epoch (Figure 1d), whereby each element tells us how much each brain region is connected to another. Then, each connectivity matrix was converted into a graph consisting of nodes (i.e., cortical ROIs) and edges (i.e., AEC value between them). Each graph was then converted into a minimum spanning tree (MST) [20], as shown in Figure 4, a sub-graph of the weighted graph, which captures the most important connections without forming close loops and which minimizes the functional cost. The cost of each link was calculated as the inverse of the connectivity value. From each MST, we computed three centrality measures that quantified the importance and influence of each node within the network, capturing distinct aspects of centrality in a network:

- **Betweenness centrality** measures how often each node appears on the shortest path between two nodes in the graph. In brain network analysis, the BC value of a brain region measures its impact on the flow of information across the brain network.
- **Closeness centrality** reflects the closeness between a node and other nodes in the brain network. In the brain network analysis, the closeness centrality of a brain region measures its indirect impact on other brain regions.
- **Eigenvector centrality** is a measure that considers both the quantity and quality of a node’s connections. In fact, it considers both the degree of the node and the degree of its neighbors.

Each centrality measure was averaged across all epochs within a specific time point in order to obtain a single averaged value per cortical region per patient. Such values were then normalized to the patient’s maximum value of relative centrality within the brain network (so that the most central region was assigned a value of 1 for each patient). Finally, we defined a new comprehensive measure of centrality, which we called “global centrality”, as the average of the normalized values of the betweenness, closeness, and eigenvector (Figure 1f). As a result, for each patient, we estimated the relative global centrality of each cortical region within the brain network, at each time point (1—before first surgery; 2—after first surgery; 3—after second surgery when present) and for each frequency band. Figure 1 provides an overview of the data analysis pipeline for the repeated surgery group. Similar pipeline was followed for the seizure-free group, whereby only one surgery was performed and only two EEGs were analyzed.

For each ROI, we also computed their post-surgical variations in centrality (i.e., post-surgical minus pre-surgical global centrality). The normalized power spectrum density (PSD) was also computed in the five frequency bands for all cortical ROIs in Brainstorm software and their post-surgical variations in relative power were estimated.

### 2.7. Statistical Analysis

To assess whether the centrality role of the untouched cortical regions within the brain network changed after surgery, we compared their global centrality between pre- and post-surgery. The Wilcoxon signed rank test was used to test the difference between pre-surgical and post-surgical global centrality separately for all the regions that were adjacent or far from the resection and separately for each frequency band. These analyses were conducted separately for the following types of surgery: (i) first successful surgery (seizure-free group); (ii) first failed surgery; (iii) repeated (second) failed surgery; (iv) repeated (second) successful surgery. Finally, the Wilcoxon rank sum test was used to compare variables between seizure-free and repeated surgery groups. Spearman’s correlation was also used to test for correlations between the patients’ ages or time intervals between EEGs and the patients’ post-surgical variation in centrality far from or adjacent to resection. We also tested whether the observed variations in global centrality of the cortical regions correlated with variations in relative power and whether the average relative centrality of far and adjacent regions correlated with each patient’s number of AEDs (at the time of the EEG). Here, *p*-values < 0.05 were considered significant. MATLAB R2020b (The MathWorks Inc., Natick, MA, USA) was used for statistical analysis.

## 3. Results

### 3.1. Patient Cohort

The repeated surgery group included nine children (4 female; age at first surgery: 5.4 (3.5–12.4) years). Three of them had a second successful surgery (age at second surgery: 6.7 (5.5–16.5) years), whereas six patients had a poor outcome after the second surgery (9.7 (2.6–12.8) years). The seizure-free group (single surgery) included 12 children (4 female; age at surgery: 14.5 (10.5–17.7) years). Table 1 reports the clinical and demographic characteristics of all included patients. Genetic testing did not play a role in the pre-surgical workup of our cohort. The first surgery consisted of a temporal resection in five patients (55%) for the repeated surgery group and in seven patients (58%) for the seizure-free group. In the repeated surgery group, we had 2 cases out of 9 (22%, patients #2, #6) with unknown causes of epilepsy (Table 1) and 3 cases out of 12 (25%, patients #11, #18, #21) in the seizure-free group; thus, since the proportion did not differ, we can exclude that this factor influenced the observed differences between groups. Age at first surgery was lower in the repeated surgery compared to the seizure-free group (*p* = 0.03). The intervals between pre- and post-surgical EEG did not differ between groups (seizure-free: 1.8 (1–3.1) years; repeated surgery: 1st 1.2 (1–1.8), *p* = 0.50; 2nd 1.2 (0.75–2.2), *p* = 0.56). No differences were observed in the percentages of the cortex that was resected during surgery between the repeated surgery group (at first surgery) and seizure-free group (5% and 3%, *p* = 0.3). No difference was seen between the repeated surgery and seizure-free group in the number of AEDs either before (*p* = 0.85) or after (1st) surgery (*p* = 0.14).

### 3.2. Functional Connectivity Changes: Pre-Surgical vs. Post-Surgical Centrality

To explore post-surgical changes in the brain network in the different groups, we compared the global centrality of the remaining regions (far and adjacent to resection) between pre- and post-surgery. Table 2 shows the results that we obtained in the five frequency bands for both adjacent and far regions.

**Figure 5:**
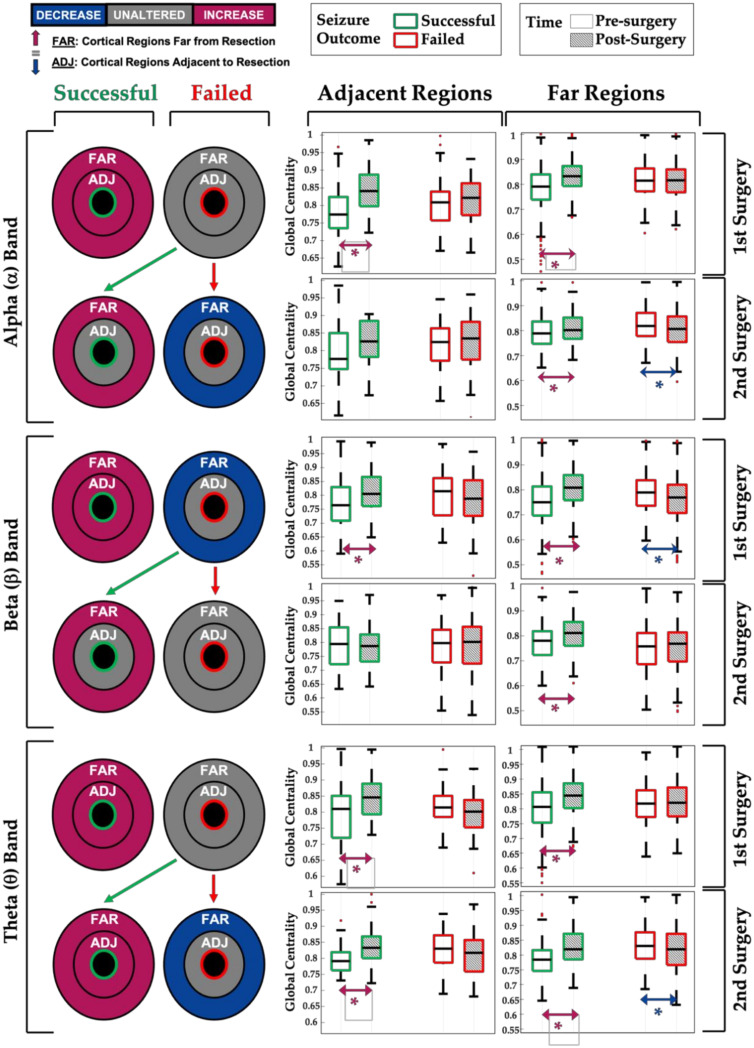
Global centrality in alpha, beta, and theta bands. For each frequency band, four circular diagrams and the corresponding boxplots are shown. For each surgery, we compared global centrality values between pre-surgery (white boxplot) and post-surgery (dashed boxplots) separately for adjacent and far regions. In the circular diagram, adjacent and far regions correspond to the two concentric sections, which are colored based on the statistical results (purple = post-surgical increase in global centrality; blue = post-surgical decrease; grey = no change). For each frequency, the top row displays the results obtained from the analysis of the first surgeries, whereby the left side shows the seizure-free group (good outcome = green), while the right side shows the repeated surgery group (poor outcome = red). The bottom row displays the results obtained from the analysis of the second surgeries (repeated surgery group only), whereby the left side shows second surgeries followed by a good outcome (green), while the right side shows second surgeries followed by a poor outcome (red). Asterisks mark significant *p*-values (<0.05).

**Figure 6:**
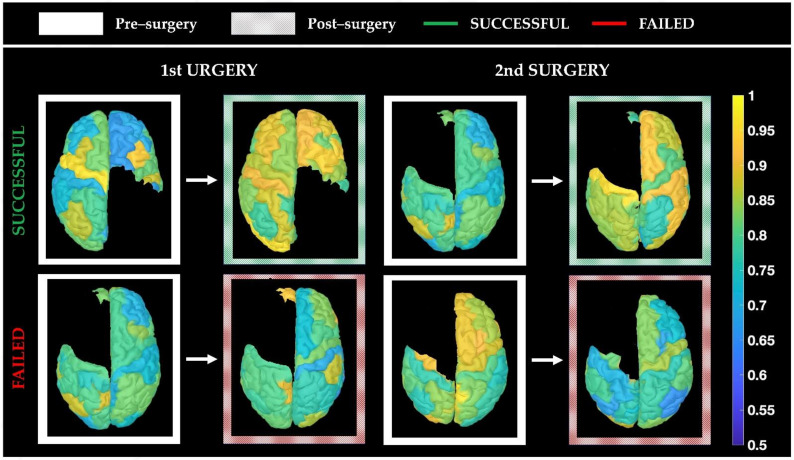
Examples of post-surgical global centrality changes in the theta band. Examples of post-surgical changes in global centrality, showing one patient image from the seizure-free group (patient 16, **top left**) and three from the repeated surgery group (patient 2, **bottom left** and **top right**; patient 4, **bottom right**). Each cortical ROI (that was not touched during surgery) is color-coded based on its global centrality value, whereby yellow indicates a high level of connectivity and blue indicates a low level of connectivity. In the successful surgery examples (**top row**), the color maps show an overall increase in post-surgical connectivity. On the other hand, in the examples of first and second failed surgeries, the color maps show no changes or decreases in connectivity.

In the seizure-free group, we found that (relative) global centrality increased after surgery in all frequency bands, both in proximity and far from resection (*p* < 0.001), with the only exception being for the adjacent regions in the gamma band (see Table 2). On the other hand, when looking at the repeated surgery group, after failed surgery, we found that theta, alpha, and beta networks did not present changes in centrality close to the resection (adjacent regions) or presented a decrease far from resection in some cases (as shown in Figure 5). In these same frequency bands, when the 2nd (repeated) surgery was successful, we observed again an increase in the global centrality (as in the seizure-free group) far from the resection (theta: *p*-value < 0.001; alpha: *p*-value = 0.01; beta: *p*-value < 0.001), as well as in its proximity for the theta band only (*p* = 0.02). Figure 5 illustrates the results for these three frequency bands. Looking at the functional network in the delta and gamma bands instead, the same post-surgical changes were seen both after a failed surgery and a successful surgery (see Table 2), nullifying a possible link with seizure outcome. Figure 6 shows four representative examples of global centrality maps before and after successful or failed surgery (first or repeated).

### 3.3. Correlation with Other Factors (Age, Time Interval, AED, Power)

We evaluated whether the intervals between EEGs or the patient’s age at the time of the pre-surgical evaluation (pre-surgical EEG) may have an influenced the observed post-surgical variation in centrality. Correlation analyses showed that: (i) averaged post-surgical variations in global centrality (for far or adjacent regions) in our cohort of patients did not depend on the time interval between EEGs (*p* > 0.05); (ii) averaged post-surgical variations in global centrality far from resection showed a negative correlation (*p* < 0.05, R < 0) with the patient’s age before surgery, meaning that the younger the child, the more positive (higher) the post-surgical variation in global centrality.

From the PSD analysis, we also found that the post-surgical variations in global centrality of the untouched cortical regions after first surgery did not correlate with their variations in relative power for all frequency bands, with the only exception being the gamma band, where a weak negative correlation was observed for both groups (R = −(0.10–0.16), *p* < 0.01)).

Finally, we did not observe any correlation in our cohort of patients between the number of AEDs (at the time of the 1st or 2nd EEG) and the averaged relative centrality of the far and adjacent regions (*p* > 0.05) in all frequency bands.

## 4. Discussion

### 4.1. Significance of the Study

Epilepsy is increasingly recognized as a neurological disorder that affects cortical network organization [36]. Several studies have shown that the brain FC and network topology can be altered through an epileptogenic focus [2,35,51]. By using advanced brain imaging techniques that combine ESI, FC, and graph theory, in the present study we investigated changes in the functional brain network of children with DRE following repeated surgery in comparison to a cohort of patients who had one successful surgery.

Repeat epilepsy surgery is a challenging proposition due to the inherent risks of subsequent surgery as well as the impetus for surgical success the second time around; children who have a first failed surgery can be judged to be eligible for re-operations, although the decision to initiate another pre-surgical evaluation is very difficult. Although re-operation rates vary between 3% and 15%, recent studies have suggested that about 30–40% of patients could benefit from a second surgery [29], further emphasizing the need for strong neurophysiological and epileptogenic network hypotheses. This was one of the few studies to investigate these challenging pediatric patients and to examine post-surgical changes in background EEG activity (independent of any epileptiform discharge). Our ultimate aim was to assess whether scalp EEG can reveal FC changes in a child’s brain network after a surgical intervention (in the proximity of or far from the resection), which seemed to be specific to this population and to their poor post-surgical seizure outcome.

### 4.2. Main Findings: Functional Connectivity Changes in the Two Cohorts of Patients

Our study suggests that advanced scalp EEG can demonstrate brain networks changes following one or two focal surgeries in children with DRE and that these changes can be linked to the seizure outcome. Our most significant findings, summarized in Figure 5, indicate that:

(i) First successful surgeries (seizure-free group) are associated with an increase in the relative centrality of the remaining (untouched) brain regions within the alpha, beta, or theta network (see Figure 5, green boxplots for 1st surgery);
(ii) Post-surgical seizure recurrence (surgery failure) on the other hand, after a first or second epilepsy surgery, is associated with a decrease or no change in the relative centrality of the remaining brain regions within the brain network (see Figure 5, red boxplots);
(iii) When the repeated (2nd) surgery is successful (i.e., is followed by seizure freedom), we observed an increase in network centrality, especially for the cortical regions far from the surgical resection (see Figure 5, green boxplots for 2nd surgery), similar to what is seen in patients who became seizure-free after their first surgery;
(iv) Network changes in the alpha, theta, and beta EEG bands seem to be associated with seizure outcome, as opposed to the changes observed in the delta and gamma bands, which did not correlate with the seizure outcome (see Table 2).

We hypothesize that the increase in relative centrality following successful surgeries may indicate a re-organization of the overall brain network towards a more uniform configuration, whereby the untouched (non-epileptogenic) regions (remaining cortex adjacent or far from resection) gain centrality within the network, likely secondary to the resection of the epileptogenic focus. Conversely, the unaltered or diminished centrality, which we observed following failed surgeries, may suggest an overall compensation effect of the untouched cortical regions aiming to isolate the persistent epileptogenic focus.

Previous fMRI [9,11,52], intracranial EEG [13,15,18], and MEG [21] studies have mostly focused on examining the connectivity of the epileptogenic focus or resected area, characterized by high local connectivity, whereby good post-surgical outcome was related to high FC of the resected brain region. In contrast, here we examined both pre-surgical and post-surgical whole-brain connectivity in children with DRE and reported how the FC of the remaining non-resected areas evolves after one or two epilepsy surgeries, depending on the outcome. This work adds to certain functional and structural connectivity studies [15,52,53] that have suggested that decreased distant connectivity in epilepsy results in a network that isolates the epileptic focus from the rest of the brain. In our cohort, removal of the epileptogenic focus was associated with increased relative connectivity in the rest of the network (because of the abolition of a highly central node), while the presence of a persistent epileptogenic focus after surgery led to a decreased or unaltered centrality role of the remaining cortical regions. Our scalp EEG results are also in line with previous MEG [21] and fMRI [10,12,14,54] studies in the field, which reported widespread decreases in connectivity in patients with focal epilepsy versus controls. Englot et al. [21] used MEG in adult patients to show that although focal epilepsy is often associated with increased local connectivity in the epileptogenic zone, it also leads to decreased long-range connectivity outside of it. Additionally, Lagarde et al. [15] using stereo-EEG found that in patients with DRE (mixed population of children and adults), before surgery non-epileptogenic areas showed low connectivity compared to the higher values exhibited for the epileptogenic and propagation zone.

Of note is also the fact that our findings are based on sleep EEG epochs containing “background activity”, free of any epileptiform. This implies that during the interictal state, even when the brain is not “spiking”, the functional brain network is reflective of a hierarchical epileptogenic organization, whereby non-epileptogenic regions do not gain a central role within the network until the epileptogenic focus is successfully removed.

### 4.3. Methodological Choices: Rationale, Pros, and Cons

We used routine scalp EEG data combined with MRI to estimate whole-brain FC. The choice of this approach was driven by its potentially high translational value since scalp EEG and MRI are the most widely used techniques to study the epileptic brain in any epilepsy center. Novel analytical methods that are EEG-based are of particular interest because scalp EEG is also potentially portable, safe, and routinely used during long-term pre-surgical investigations.

The main challenge of using scalp EEG is that the potentials recorded at the electrode level are subject to volume conduction (are attenuated and distorted before reaching the scalp electrodes). Electric source imaging (ESI) helps overcoming this problem by reconstructing sparsely sampled sensor signals on the scalp into the three-dimensional brain space. ESI basically combines EEG and MRI information of the patient to estimate the underlying brain activity that generates the recorded EEG [31,45,46]. As Figure 2 shows, in this study we used ESI to calculate the cortical activity (we reconstructed beamformer-based time series from 68 anatomically-defined cortical ROIs) and then applied FC analysis to analyze the relationships between these cortical sources. The consistency of our findings with previous works and the ability to highlight different behaviors in our two different cohorts of patients suggest that this approach allows to monitor how the functional brain network evolves in children with DRE at the cortical level (see Figure 1 and Figure 2).

It is important to acknowledge that ESI-based approaches to estimate FC are inherently limited by the fact that source estimates are highly spatially correlated and that this can cause the resemblance of connectivity between regions (because of spatial leakage of the estimated sources into neighbors) [50,55]. Many methods exist for FC estimation, and some are more sensitive than others to this spatial leakage confound. We chose the AEC (amplitude envelope correlation), as implemented in the Brainstorm open-source software, after orthogonalization correction, which removes zero-lag signal overlaps that could be attributed to spatial leakage effects. This choice was based on: (a) the studies by Brookes et al. [49] and Hipp et al. [48], who introduced this novel measure to overcome limitations due to the limited spatial resolution of electrophysiological measures and demonstrated how it estimates functional networks that are very similar to those from fMRI; (b) the technical note from Colclough et al. [50] showing how this was the most consistent and repeatable connectivity measure for resting-state studies in the source space. It is also true that using FC methods, one can estimate a strong connection between two ROIs only because they both have high power signals. For this reason, here we also examined the relative power in the five frequency bands for each cortical ROI and tested their correlation with their FC estimate; we did not find any positive correlation, excluding the likelihood of a systematic bias in our cohort.

We must also mention that non-invasive recording techniques with a higher number of sensors [56], such as high-density EEG (>64 channels) or MEG [47,57], allow a more precise reconstruction of the network through smaller ROIs (also called virtual sensors). Most clinics still lack the equipment or resources to perform MEG or EEG monitoring with >64 electrodes: thus, while our proposed approach can be significantly enhanced in terms of localizing value, it paves the way for an easily implementable approach in clinics. Intracranial EEG also offers a significant improvement in the spatial resolution and signal-to-noise ratio compared to routine scalp EEG, although intracranial EEG studies are inherently limited: (i) to the area of electrode implantation, not permitting whole-brain connectivity analysis; (ii) by the invasiveness of the technique, which can be used as a pre-surgical tool only for selected patients and not as a post-surgical technique to monitor the disease.

In summary, the methodological EEG-based approach that we proposed here provides an estimation of the functional brain network thatmay further the utility of traditional scalp EEG reading, which is typically limited to the interpretation of epileptiform discharges at the scalp level. Our findings must be interpreted carefully considering the above-mentioned factors that can influence the analysis (FC and ESI method, type of epilepsy, AEDs, resting-state network and graph analysis); nevertheless, they contribute to paving the way to bring source space connectivity into clinical practice.

### 4.4. Limitations and Future Directions

Our pediatric cohort was heterogeneous in terms of the type and location of resection (Table 1); although we could not control for this factor (due to the low sample size), results for the extent of the resected brain and type of surgery (temporal vs. extratemporal) did not differ between our seizure-free and repeated surgery group. Eventually, analysis of a large cohort of patients that allows correction for possible confounders such as pathology, seizure frequency, age at epilepsy onset, and time between resection and post-surgical EEG should be implemented to investigate these interactions.

The low sample size did not allow us to investigate the clinical value of the approach at the individual patient level and whether the proposed parameters are crucial to the surgical work-up. Larger prospective studies are recommended to test the relevance of the proposed EEG parameters in the pre-surgical work-up for re-operation. It is also important to note that our findings may depend on the choice of specific source localization and FC methods; a comparison between ESI and FC methods was out of the scope of our study but is warranted in the future.

Our seizure-free patients were older than the repeated surgery ones; although we did not control for this factor, we found an opposite correlation between a patient’s age and their global centrality variation after surgery, reducing the possibility that our findings (positive variation of global centrality after surgery in the seizure-free but not repeated surgery patients) were due to such an age difference. Furthermore, to exclude the possibility that our findings were biased by the younger ages of the repeated surgery group at first surgery, we tested whether the same trends were seen when the two very young (<3 years old) patients (#3 and #8) were not included in the analysis. This confirmed the same statistical trend seen following the failed surgery, as we observed no changes (*p* > 0.05) in post-surgical connectivity in alpha, theta, and beta band both for far and adjacent areas and a decrease for the beta band in far areas (*p* < 0.001). Our data suggest that the reported findings are robust despite the age differences.

Another possible limitation is the influence of AEDs [58]. Patients in our series presented different types and number of AEDs at the time of recordings. Although we could not control for or investigate the effect of this variable on the post-surgical network changes, we observed that the number of AEDs at the time of the EEG did not correlate with the FC estimates adjacent of far from resection in any frequency, reducing the possibility of a bias introduced by this confounder on our findings.

## 5. Conclusions

This study presents the first evidence that EEG-based FC analysis at the source level can reveal epilepsy network changes following one or two epilepsy surgery in children with DRE. A decrease or no change in the centrality of the untouched brain regions within the brain network is seen for post-surgical seizure recurrence (meaning surgery failure). On the other hand, an increase in the overall centrality of the untouched brain regions is observed for successful surgeries. In particular, FC changes in the alpha, theta and beta EEG bands seem to be related to surgery outcome. We can speculate that unaltered or diminished centrality following epilepsy surgery is a consequence of an overall compensation effect, whereby the untouched cortical regions aim to isolate the persistent epileptogenic focus. An overall increase in FC after first surgery possibly indicates a re-organization of the overall brain network, whereby the untouched (non-epileptogenic) regions gain centrality within the network, since the epileptogenic focus has been successfully removed.

EEG-based FC analysis at the cortical level can provide information on the impacts of epilepsy surgery on brain networks. The low sample size and heterogenicity of our cohort did not allow us to demonstrate the value of our measures for individual patient care. Further studies are needed to investigate whether the proposed EEG measures are crucial to pre-surgical workup and can aid outcome prediction after re-operation.

## Figures and Tables

**Table 1:** Clinical characteristics of our cohort.

		Age (Y)					No. AED			Resection (Side Lobe)		
			Surgery				EEG			Surgery		
Pt/Sex	Gr.	Epi. Ons	1st	2nd	Etiology	Pathology (Histology)	1st	2nd	3rd	1st	2nd	Engel ^a^
1/Fe	RS	3	5.3	6.7	FCD	FCD 2A	3	2	1	L-T	L-T	I
2/M	RS	4	16.8	19.8	Unknown	Gliosis	2	3	0	L-F	L-F	I
3/Fe	RS	0.7	1.7	2.5	Polymicrogyria	FCD 2A, Gliosis, Fused Sulci, WM heteropia	3	3	2	L-F	L-F	III
4/M	RS	1.5	5.4	7.3	FCD	FCD 2B, Gliosis	4	4	4	L-F	L-F	II
5/M	RS	10	15.5	17.4	Low grade glioma + developmental venous anomaly	Low grade glioma, dysplastic features	1	2	2	R-T	R-C	II
6/Fe	RS	0.8	11.3	12.8	Unknown	Oligodendroglial hyperplasia	2	2	2	R-T	R-T	IV
7/M	RS	10	11.3	12.0	Sphenoidal encephalocele	Dysplastic features, Gliosis	2	2	3	L-T	L-T	II
8/Fe	RS	0	2.1	2.6	TSC	Consistent w/TSC	2	3	2	R-F	R-PF	IV
9/M	RS	3	4.0	5.1	FCD	FCD 2A, MST	2	2	1	R-T	R-T	I
10/M	SF	9	17.4	n/a	Neoplasm	Ganglioma, FCD 1	3	3	na	L-T	na	I
11/Fe	SF	16	18.2	n/a	Unknown	Gliosis	2	1	na	L-T	na	I
12/M	SF	5	5.5	n/a	Lesion suggestive of DNET	Angiocentric glioma	4	0	na	L-C	na	I
13/M	SF	1.5	12.5	n/a	MTS	FCD 2A	3	2	na	L-T	na	I
14/M	SF	8	9.8	n/a	Polymicrogyria	Mild dysplastic features	2	3	na	L-P	na	I
15/M	SF	6	14.8	n/a	FCD	na ^b^	3	3	na	R-F	na	I
16/M	SF	8	10.0	n/a	Posterior cerebralartery infarct (PCA)	Consistent w/PCA encephalomalacia, FCD 2D	2	2	na	R-O	na	I
17/Fe	SF	17	21	n/a	TBI	Consistent w/TBI	2	3	na	R-T	na	I
18/Fe	SF	12	18	n/a	Unknown	MTS, gliosis, oligodendroglial hyperplasia	1	0	na	L-T	na	I
19/M	SF	13	14.2	n/a	Low grade tumor	DNET	5	2	na	L-T	na	I
20/Fe	SF	0.6	16.1	n/a	MTS + FCD	MST, FCD 2A	2	1	na	L-T	na	I
21/M	SF	5	10.8	n/a	Unknown	na ^b^	1	1	na	R-P	na	I

**^a^** For repeated surgery (RS) group, the reported Engel scale outcome is relative to their 2nd surgery. **^b^** Histological exam was not performed (laser ablation cases). Pt = Patient; Y = Years; M = Male; Fe = Female; Gr = Group; RS = Repeated surgery; SF = Seizure-free; Epi. Onset = Age at epilepsy onset; Surg = Surgery; n/a = Not applicable; AED = Anti-epileptic drugs; L = Left; R = Right; F = Frontal; T = Temporal; P = Parietal; O = Occipital; C = Central; FCD = Focal cortical dysplasia; TSC = Tuberous sclerosis complex; DNET = Dysembryoplastic neuroepithelial tumor; TBI = Traumatic brain injury; WM = White matter; MTS = Mesial temporal sclerosis.

**Table 2:** Analysis of post-surgical changes in the global centrality of far and adjacent regions.

		1st Surgery		2nd (Repeated) Surgery	
Frequency	Areas	Successful (SF Group)	Failed (RS Group)	Successful (RS Group)	Failed (RS Group)
Delta (δ)	FAR	*p* < 0.001 * (increase)	*p* < 0.001 * (increase)	*p* = 0.3 (no change)	*p* < 0.001 * (decrease)
	ADJACENT	*p* < 0.001 * (increase)	*p* = 0.04 * (decrease)	*p* = 0.5 (no change)	*p* = 0.2 (no change)
Theta (θ)	FAR	*p* < 0.001 * (increase)	*p* = 0.1 (no change)	*p* < 0.001 * (increase)	*p* = 0.01 * (decrease)
	ADJACENT	*p* < 0.001 * (increase)	*p* = 0.06 (no change)	*p* = 0.02 * (increase)	*p* = 0.4 (no change)
Alpha (α)	FAR	*p* < 0.001 * (increase)	*p* = 0.8 (no change)	*p* = 0.01 * (increase)	*p* = 0.004 * (decrease)
	ADJACENT	*p* < 0.001 * (increase)	*p* = 0.4 (no change)	*p* = 0.2 (no change)	*p* = 0.4 (no change)
Beta (β)	FAR	*p* < 0.001 * (increase)	*p* < 0.001 * (decrease)	*p* < 0.001 * (increase)	*p* = 0.3 (no change)
	ADJACENT	*p* < 0.001 * (increase)	*p* = 0.2 (no change)	*p* = 0.8 (no change)	*p* = 0.4 (no change)
Gamma (γ)	FAR	*p* = 0.001 * (increase)	*p* < 0.001 * (decrease)	*p* = 0.4 (no change)	*p* < 0.01 * (increase)
	ADJ	*p* = 0.06 (no change)	*p* = 0.6 (no change)	*p* = 0.009 * (decrease)	*p* = 0.9 (no change)

RS = Repeated surgery; SF = Seizure-Free; *p* = *p*-value (Wilcoxon signed rank test); * *p*-value statistically significant.

## Data Availability

The data presented in this study are available on request from the corresponding author. The data are not publicly available due to privacy restrictions.

## References

[1] Sinha N., Dauwels J., Kaiser M., Cash S.S., Westover M.B., Wang Y., Taylor P.N. (2017). Predicting neurosurgical outcomes in focal epilepsy patients using computational modelling. Brain.

[2] Van Diessen E., Diederen S.J.H., Braun K.P.J., Jansen F.E., Stam C.J. (2013). Functional and structural brain networks in epilepsy: What have we learned?. Epilepsia.

[3] Foit N.A., Bernasconi A., Bernasconi N. (2020). Functional Networks in Epilepsy Presurgical Evaluation. Neurosurg. Clin. N. Am..

[4] Stefan H., da Silva F.H.L. (2013). Epileptic neuronal networks: Methods of identification and clinical relevance. Front. Neurol..

[5] Haneef Z., Chiang S. (2014). Clinical correlates of graph theory findings in temporal lobe epilepsy. Seizure.

[6] Spencer S.S. (2002). Neural Networks in Human Epilepsy: Evidence of and Implications for Treatment. Clin. Res..

[7] Wang Y., Wang X., Mo J.J., Sang L., Zhao B.T., Zhang C., Hu W.H., Zhang J.G., Shao X.Q., Zhang K. (2019). Symptomatogenic zone and network of oroalimentary automatisms in mesial temporal lobe epilepsy. Epilepsia.

[8] Varela F., Lachaux J.P., Rodriguez E., Martinerie J. (2001). The brainweb: Phase synchronization and large-scale integration. Nat. Rev. Neurosci..

[9] Liao W., Zhang Z., Pan Z., Mantini D., Ding J., Duan X., Luo C., Lu G., Chen H. (2010). Altered functional connectivity and small-world in mesial temporal lobe epilepsy. PLoS ONE.

[10] Luo C., Qiu C., Guo Z., Fang J., Li Q., Lei X., Xia Y., Lai Y., Gong Q., Zhou D. (2012). Disrupted functional brain connectivity in partial epilepsy: A resting-state fMRI study. PLoS ONE.

[11] Haneef Z., Lenartowicz A., Yeh H.J., Levin H.S., Engel J., Stern J.M. (2014). Functional connectivity of hippocampal networks in temporal lobe epilepsy. Epilepsia.

[12] Haneef Z., Lenartowicz A., Yeh H.J., Engel J., Stern J.M. (2014). Network analysis of the default mode network using functional connectivity MRI in temporal lobe epilepsy. J. Vis. Exp..

[13] Holmes M., Folley B.S., Sonmezturk H.H., Gore J.C., Kang H., Abou-Khalil B., Morgan V.L. (2014). Resting state functional connectivity of the hippocampus associated with neurocognitive function in left temporal lobe epilepsy. Hum. Brain Mapp..

[14] Maneshi M., Vahdat S., Fahoum F., Grova C., Gotman J. (2014). Specific resting-state brain networks in mesial temporal lobe epilepsy. Front. Neurol..

[15] Lagarde S., Roehri N., Lambert I., Trebuchon A., McGonigal A., Carron R., Scavarda D., Milh M., Pizzo F., Colombet B. (2018). Interictal stereotactic-EEG functional connectivity in refractory focal epilepsies. Brain.

[16] Marino A.C., Yang G.J., Tyrtova E., Wu K., Zaveri H.P., Farooque P., Spencer D.D., Bandt S.K. (2019). Resting state connectivity in neocortical epilepsy: The epilepsy network as a patient-specific biomarker. Clin. Neurophysiol..

[17] Quraan M.A., McCormick C., Cohn M., Valiante T.A., McAndrews M.P. (2013). Altered Resting State Brain Dynamics in Temporal Lobe Epilepsy Can Be Observed in Spectral Power, Functional Connectivity and Graph Theory Metrics. PLoS ONE.

[18] Bettus G., Wendling F., Guye M., Valton L., Régis J., Chauvel P., Bartolomei F. (2008). Enhanced EEG functional connectivity in mesial temporal lobe epilepsy. Epilepsy Res..

[19] Adebimpe A., Aarabi A., Bourel-Ponchel E., Mahmoudzadeh M., Wallois F. (2016). EEG resting state functional connectivity analysis in children with benign epilepsy with centrotemporal spikes. Front. Neurosci..

[20] Van Dellen E., Douw L., Hillebrand A., de Witt Hamer P.C., Baayen J.C., Heimans J.J., Reijneveld J.C., Stam C.J. (2014). Epilepsy surgery outcome and functional network alterations in longitudinal MEG: A minimum spanning tree analysis. Neuroimage.

[21] Englot D.J., Hinkley L.B., Kort N.S., Imber B.S., Mizuiri D., Honma S.M., Findlay A.M., Garrett C., Cheung P.L., Mantle M. (2015). Global and regional functional connectivity maps of neural oscillations in focal epilepsy. Brain.

[22] Aydin Ü., Pellegrino G., Ali O.B.K.B., Abdallah C., Abdallah C., Dubeau F., Lina J.M., Lina J.M., Kobayashi E., Grova C. (2020). Magnetoencephalography resting state connectivity patterns as indicatives of surgical outcome in epilepsy patients. J. Neural Eng..

[23] De Palma L., De Benedictis A., Specchio N., Marras C.E. (2020). Epileptogenic Network Formation. Neurosurg. Clin. N. Am..

[24] Téllez-Zenteno J.F., Dhar R., Wiebe S. (2005). Long-term seizure outcomes following epilepsy surgery: A systematic review and meta-analysis. Brain.

[25] Spencer S., Huh L. (2008). Outcomes of epilepsy surgery in adults and children. Lancet Neurol..

[26] Ramantani G., Strobl K., Stathi A., Brandt A., Schubert-Bast S., Wiegand G., Korinthenberg R., Stephani U., Van Velthoven V., Zentner J. (2013). Reoperation for refractory epilepsy in childhood: A second chance for selected patients. Neurosurgery.

[27] Shaver E.G., Harvey A.S., Morrison G., Prats A., Jayakar P., Dean P., Duchowny M. (1997). Results and complications after reoperation for failed epilepsy surgery in children. Pediatr. Neurosurg..

[28] Muthaffar O., Puka K., Rubinger L., Go C., Snead O.C., Rutka J.T., Widjaja E. (2017). Reoperation after failed resective epilepsy surgery in children. J. Neurosurg. Pediatr..

[29] Grote A., Witt J.A., Surges R., Von Lehe M., Pieper M., Elger C.E., Helmstaedter C., Ormond D.R., Schramm J., Delev D. (2016). A second chance-reoperation in patients with failed surgery for intractable epilepsy: Long-term outcome, neuropsychology and complications. J. Neurol. Neurosurg. Psychiatry.

[30] Schoffelen J.M., Gross J. (2009). Source connectivity analysis with MEG and EEG. Hum. Brain Mapp..

[31] Tamilia E., AlHilani M., Tanaka N., Tsuboyama M., Peters J.M., Grant P.E., Madsen J.R., Stufflebeam S.M., Pearl P.L., Papadelis C. (2019). Assessing the localization accuracy and clinical utility of electric and magnetic source imaging in children with epilepsy. Clin. Neurophysiol..

[32] Tamilia E., Dirodi M., Alhilani M., Grant P.E., Madsen J.R., Stufflebeam S.M., Pearl P.L., Papadelis C. (2020). Scalp ripples as prognostic biomarkers of epileptogenicity in pediatric surgery. Ann. Clin. Transl. Neurol..

[33] Ricci L., Tamilia E., Alhilani M., Alter A., Scott Perry Μ., Madsen J.R., Peters J.M., Pearl P.L., Papadelis C. (2021). Source imaging of seizure onset predicts surgical outcome in pediatric epilepsy. Clin. Neurophysiol..

[34] Zijlmans M., Zweiphenning W., van Klink N. (2019). Changing concepts in presurgical assessment for epilepsy surgery. Nat. Rev. Neurol..

[35] Alstott J., Breakspear M., Hagmann P., Cammoun L., Sporns O. (2009). Modeling the impact of lesions in the human brain. PLoS Comput. Biol..

[36] Kramer M.A., Cash S.S. (2012). Epilepsy as a disorder of cortical network organization. Neuroscientist.

[37] Van Diessen E., Zweiphenning W.J.E.M., Jansen F.E., Stam C.J., Braun K.P.J., Otte W.M. (2014). Brain network organization in focal epilepsy: A systematic review and meta-analysis. PLoS ONE.

[38] Bullmore E., Sporns O. (2009). Complex brain networks: Graph theoretical analysis of structural and functional systems. Nat. Rev. Neurosci..

[39] Van Straaten E.C.W., Stam C.J. (2013). Structure out of chaos: Functional brain network analysis with EEG, MEG, and functional MRI. Eur. Neuropsychopharmacol..

[40] Lang E.W., Tomé A.M., Keck I.R., Górriz-Sáez J.M., Puntonet C.G. (2012). Brain connectivity analysis: A short survey. Comput. Intell. Neurosci..

[41] Dale A.M., Fischl B., Sereno M.I. (1999). Cortical surface-based analysis: I. Segmentation and surface reconstruction. Neuroimage.

[42] Zöllei L., Iglesias J.E., Ou Y., Grant P.E., Fischl B. (2020). Infant FreeSurfer: An automated segmentation and surface extraction pipeline for T1-weighted neuroimaging data of infants 0–2 years. Neuroimage.

[43] Desikan R.S., Ségonne F., Fischl B., Quinn B.T., Dickerson B.C., Blacker D., Buckner R.L., Dale A.M., Maguire R.P., Hyman B.T. (2006). An automated labeling system for subdividing the human cerebral cortex on MRI scans into gyral based regions of interest. Neuroimage.

[44] Tadel F., Baillet S., Mosher J., Pantazis D., Leahy R. (2011). Brainstorm: A user-friendly application for MEG/EEG analysis. Comput. Intell. Neurosci..

[45] Ntolkeras G., Tamilia E., AlHilani M., Bolton J., Ellen Grant P., Prabhu S.P., Madsen J.R., Stufflebeam S.M., Pearl P.L., Papadelis C. (2021). Presurgical accuracy of dipole clustering in MRI-negative pediatric patients with epilepsy: Validation against intracranial EEG and resection. Clin. Neurophysiol..

[46] Ricci L., Assenza G., Pulitano P., Simonelli V., Vollero L., Lanzone J., Mecarelli O., Di Lazzaro V., Tombini M. (2021). Measuring the effects of first antiepileptic medication in Temporal Lobe Epilepsy: Predictive value of quantitative-EEG analysis. Clin. Neurophysiol..

[47] Tamilia E., Matarrese M.A.G., Ntolkeras G., Grant P.E., Madsen J.R., Stufflebeam S.M., Pearl P.L., Papadelis C. (2021). Noninvasive Mapping of Ripple Onset Predicts Outcome in Epilepsy Surgery. Ann. Neurol..

[48] Hipp J.F., Hawellek D.J., Corbetta M., Siegel M., Engel A.K. (2012). Large-scale cortical correlation structure of spontaneous oscillatory activity. Nat. Neurosci..

[49] Brookes M.J., Woolrich M., Luckhoo H., Price D., Hale J.R., Stephenson M.C., Barnes G.R., Smith S.M., Morris P.G. (2011). Investigating the electrophysiological basis of resting state networks using magnetoencephalography. Proc. Natl. Acad. Sci. USA.

[50] Colclough G.L., Woolrich M.W., Tewarie P.K., Brookes M.J., Quinn A.J., Smith S.M. (2016). How reliable are MEG resting-state connectivity metrics?. Neuroimage.

[51] Van Dellen E., Hillebrand A., Douw L., Heimans J.J., Reijneveld J.C., Stam C.J. (2013). Local polymorphic delta activity in cortical lesions causes global decreases in functional connectivity. Neuroimage.

[52] Luo C., An D., Yao D., Gotman J. (2014). Patient-specific connectivity pattern of epileptic network in frontal lobe epilepsy. NeuroImage Clin..

[53] Nissen I.A., van Klink N.E.C., Zijlmans M., Stam C.J., Hillebrand A. (2016). Brain areas with epileptic high frequency oscillations are functionally isolated in MEG virtual electrode networks. Clin. Neurophysiol..

[54] Voets N.L., Beckmann C.F., Cole D.M., Hong S., Bernasconi A., Bernasconi N. (2012). Structural substrates for resting network disruption in temporal lobe epilepsy. Brain.

[55] Palva S., Palva J.M. (2012). Discovering oscillatory interaction networks with M/EEG: Challenges and breakthroughs. Trends Cogn. Sci..

[56] Hedrich T., Pellegrino G., Kobayashi E., Lina J.M., Grova C. (2017). Comparison of the spatial resolution of source imaging techniques in high-density EEG and MEG. Neuroimage.

[57] Juárez-Martinez E.L., Nissen I.A., Idema S., Velis D.N., Hillebrand A., Stam C.J., van Straaten E.C.W. (2018). Virtual localization of the seizure onset zone: Using non-invasive MEG virtual electrodes at stereo-EEG electrode locations in refractory epilepsy patients. NeuroImage Clin..

[58] Pellegrino G., Mecarelli O., Pulitano P., Tombini M., Ricci L., Lanzone J., Brienza M., Davassi C., Di Lazzaro V., Assenza G. (2018). Eslicarbazepine Acetate Modulates EEG Activity and Connectivity in Focal Epilepsy. Front. Neurol..

